# Incidence of chemotherapy-induced amenorrhea associated with epirubicin, docetaxel and navelbine in younger breast cancer patients

**DOI:** 10.1186/1471-2407-10-281

**Published:** 2010-06-11

**Authors:** Wen-Bin Zhou, Hong Yin, Xiao-An Liu, Xiao-Ming Zha, Lin Chen, Jun-Cheng Dai, Ai-di Tao, Ling Chen, Jing-Jing Ma, Li-Jun Ling, Shui Wang

**Affiliations:** 1 Department of Breast Surgery, The First Affiliated Hospital of Nanjing Medical University, 300 Guangzhou Road, 210029 Nanjing, PR China; 2 Department of Breast Surgery, Nanjing Maternity and Child Health Care Hospital of Nanjing Medical University, Nanjing, PR China; 3 Department of Epidemiology and Biostatistics, Nanjing Medical University School of Public Health, 140 Hanzhong Road, 210029 Nanjing, PR China

## Abstract

**Background:**

The rates of chemotherapy-induced amenorrhea (CIA) associated with docetaxel-based regimens reported by previous studies are discordant. For navelbine-based chemotherapies, rates of CIA have seldom been reported.

**Methods:**

Of 170 premenopausal patients recruited between January 2003 and September 2008, 78 were treated with fluorouracil plus epirubicin and cyclophosphamide (FEC), 66 were treated with docetaxel plus epirubicin (TE), and 26 were treated with navelbine plus epirubicin (NE). Patient follow-up was carried up every 3-4 months during the first year, then every 9-12 months during subsequent years.

**Results:**

In univariate analysis, the rates of CIA were 44.87% for the FEC regimen, 30.30% for the TE regimen and 23.08% for the NE regimen (*P *= 0.068). Significant differences in the rates of CIA were not found between the FEC and TE treatment groups (*P *> 0.05), but were found between the FEC and NE treatment groups (*P *< 0.05). Furthermore, no significant differences were found between the TE and NE regimens (*P *> 0.05). Tamoxifen use was a significant predictor for CIA (*P *= 0.001), and age was also a significant predictor (*P *< 0.001). In multivariate analysis, age (*P *< 0.001), the type of chemotherapy regimens (*P *= 0.009) and tamoxifen use (*P *= 0.003) were all significant predictors.

**Conclusions:**

Age and administration of tamoxifen were found to be significant predictive factors of CIA, whereas docetaxel and navelbine based regimens were not associated with higher rates of CIA than epirubicin-based regimen.

## Background

For approximately 25% of women with breast cancer in the United States, their diagnosis was made prior to the onset of menopause [1-3]. Adjuvant chemotherapy has been shown to prolong disease-free survival and overall survival in younger breast cancer patients [4,5]. However, adjuvant chemotherapy can also induce many adverse effects. These adverse effects include gastrointestinal reactions, myelosuppression, and suppression of ovarian function [3,6]. Therefore, for younger women who are concerned about preserving their fertility, the possibilities of premature menopause and infertility should be considered in decisions regarding adjuvant chemotherapy [7].

The main factors contributing to chemotherapy-induced amenorrhea (CIA) are patient age, and dosage and schedule of chemotherapy [8]. A favorable effect of CIA has been observed for disease outcome in premenopausal women [9], and a previous study showed pregnancies following the diagnosis of breast cancer did not adversely affect the prognosis of early-stage breast cancer [10]. Another study reported the risk of CIA associated with adjuvant chemotherapies involving alkylating agents or anthracyclines to range from 53-89% [11]. Although docetaxel is widely used in adjuvant chemotherapies prescribed, studies show discordant results in the rates of CIA associated with docetaxel-based regimens. For navelbine-based chemotherapies, rates of CIA have seldom been reported.

In the present study, we evaluated the impact of docetaxel and navelbine in anthracycline-based chemotherapy regimens on the incidence of CIA for premenopausal patients diagnosed with breast cancer. The impact of endocrine therapy (i.e. tamoxifen) following chemotherapy on the incidence of CIA was also assessed.

## Methods

### Patients

Between January 2003 and September 2008, 201 premenopausal patients diagnosed with early breast cancer were recruited at the First Affiliated Hospital of Nanjing Medical University and Nanjing Maternity and Child Health Care Hospital of Nanjing Medical University, Nanjing, Jiangsu, China. The following criteria were the basis for exclusion of patient data from this retrospective study: (1) treatment with luteinizing hormone releasing hormone (LHRH) agonists; (2) bilateral oophorectomy; (3) recurrent disease within 12 months; (4) stage IV breast cancer and (5) receiving chemotherapy previously. Since 13 of the 201 patients received bilateral oophorectomy, 7 had been treated with LHRH, 7 were lost during follow-up, and 4 had recurrent disease within 12 months, patient data from only 170 patients were included in this descriptive study and analyzed.

Information about CIA and will of pregnancy were collected by telephone. All patients provided oral consent for their clinical data to be reviewed by us. This study has been performed with the approval of the ethics committee of the First Affiliated Hospital of Nanjing Medical University and Nanjing Maternity and Child Health Care Hospital of Nanjing Medical University, and this study was in compliance with the Helsinki Declaration. CIA was defined as the cessation of menses for 12 consecutive months after the end of chemotherapy for evaluating the destruction of the ovarian reserve [12,13], and menopause was defined according to National Comprehensive Cancer Network (NCCN) guidelines. Additional clinical information collected for this non-randomized study included age, tumor stage and size, nodal stage, number of nodes involved and removed, hormone receptor status, status of other biological variables (i.e. HER2, p53), surgical treatment (modified radical mastectomy, breast-conserving surgery), radiotherapy administered, and adjuvant chemotherapy or endocrine therapy received. Rates of CIA were analyzed according to age, chemotherapy regimen, and tamoxifen use.

### Treatment

Adjuvant chemotherapy regimens were determined based on guidelines in our country and included: (1) for the FEC regimen, 5-fluorouracil (500 mg/m^2^), epirubicin (75 mg/m^2^), cyclophosphamide (500 mg/m^2^) on day 1 every 3 weeks for six cycles; (2) for the TE regimen, docetaxel (75 mg/m^2^), epirubicin (60 mg/m^2^) on day 1 every 3 weeks for six cycles; (3) for the NE regimen, navelbine (25 mg/m^2^) on days 1 and 8, epirubicin (60 mg/m^2^) on day 1 every 3 weeks for six cycles. To reach 100% dose, all the patients treated with TE regimen were granulocyte colony stimulating factor (G-CSF) supported, and the patients treated with the other two regimens were also G-CSF supported in the case of low white blood cell count (WBC, <3.0 × 10^9^/L on day 10 after chemotherapy). Following adjuvant chemotherapy, tamoxifen was additionally prescribed if patients were positive for estrogen receptor (ER) and/or progesterone receptor (PR). A subset of patients switched to the aromatase inhibitor after at least 2-year tamoxifen use, and some patients received radiotherapy according to NCCN guidelines. Patient follow-up was every 3-4 months for the first year, then every 9-12 months during subsequent years. The median follow-up time from initiation of chemotherapy was 38.5 months (range, 17 - 83 months).

### Statistical analysis

Median, percentiles, and range were analyzed for each continuous variable. The candidate explanatory variables in the univariate analyses of CIA onset were: age at diagnosis, chemotherapy regimen, and use of tamoxifen. Fisher's exact test was used for univariate analyses and logistic regression was used for multivariate analysis. All *P*-values were two-tailed with 5% significance levels. All statistical analyses were performed using STATA version 9.2 (Computer Resource Center, America).

## Results

A total of 170 patients diagnosed with breast cancer prior to the onset of menopause were included in this analysis, 78 were treated with a FEC regimen, 66 with a TE regimen, and 26 with a NE regimen. The mean age of these patients was 42.48 y (range, 23-53 y).

Patient characteristics according to the chemotherapy regimen received are shown in Table 1. No significant differences were found for the three treatment groups regarding their mean age, pathology, or use of tamoxifen. However, an increased proportion of stage II and III cancers were associated with TE and NE regimens (*P *< 0.001).

**Table 1:** Patient characteristics by chemotherapy regimen received

Patient Characteristics	Regimen of chemotherapy			*P*-value
	FEC (n = 78)	TE (n = 66)	NE (n = 26)	
Age at diagnosis, y				

Mean	42.72	41.62	43.96	0.20
SD	5.01	6.42	6.57	

Pathology				

IDC	70	59	23	1.00
Other	8	7	3	

Stage at diagnosis				

I	30 (38.46%)		4 (15.38%)	< 0.001
II	48 (61.54%)	36 (54.55%)	15 (57.69%)	
III		30 (45.45%)	7 (26.92%)	

Tamoxifen use, n (%)				

Yes	55 (70.51%)	44 (66.67%)	19 (73.08%)	0.832
No	23 (29.49%)	22 (33.33%)	7 (26.92%)	

*SD *standard deviation; *IDC *infitrating ductal carcinoma

### Univariate predictors of CIA

The rate of CIA determined for the 170 patients was 35.88%, and most amenorrhea came up after the second or third cycle of chemotherapy. The rates of CIA according to clinical variables are shown in Table 2. Incidence of CIA was independently analyzed for patients 40 y and younger versus patients older than 40 y. For these two groups, the rates of CIA were 12.70% and 49.53%, respectively (*P *< 0.001). It was also found that more patients 40 y or younger experienced temporary amenorrhea (data not shown) and had more regular menstruation cycles.

**Table 2:** Univariate analysis of CIA

variable	CIA
**Age at diagnosis, y**	

≤40 y	8/63 (12.70%)
>40 y	53/107 (49.53%)
*P*-value	<0.001

**Chemotherapy regimen**	

FEC	35/78 (44.87%)
TE	20/66 (30.30%)
NE	6/26 (23.08%)
*P*-value	0.068

**Tamoxifen use**	

Yes	52/117 (44.44%)
No	9/53 (16.98%)
*P*-value	0.001

The impact of chemotherapy regimens on the incidence of CIA was also analyzed. Rates of CIA were 44.87%, 30.30% and 23.08% for the FEC, TE and NE regimen groups, respectively (*P *= 0.068). Significant differences in the rates of CIA were not found between the FEC and TE treatment groups (*P *> 0.05), but were found between the FEC and NE treatment groups (*P *= 0.049). Furthermore, no significant differences were found between the TE and NE regimens (*P *> 0.05). More patients experienced temporary amenorrhea for TE regimen (data not shown). For patients receiving tamoxifen, they were more likely to become amenorrheic (*P *= 0.001). And for patients not receiving tamoxifen, more of them had temporary amenorrhea (data not shown).

### Multivariate analysis of CIA

The significant factors for CIA were age (*P *< 0.001), the type of chemotherapy regimens (*P *= 0.009) and tamoxifen use (*P *= 0.003) in multivariate analysis (Table 3). When age and tamoxifen use were adjusted, the rate of CIA for NE regimen was lower than that for FEC regimen (*P *= 0.016), but no significant differences were found between TE and FEC regimens (*P *= 0.088).

**Table 3:** Multivariate analysis of CIA

Multivariate	OR(95% CI)	*P*-value of CIA
Age (≤40 y vs. >40 y*)	0.14(0.06-0.34)	<0.001
Chemotherapy (FEC* vs. TE vs. NE)	0.51(0.30-0.84)	0.009
Tamoxifen use (yes vs. no*)	3.69(1.54-8.83)	0.003

*Reference category

### Analysis of patients' preference for children

All patients were asked whether they had considered the option to have children, or additional children, following treatment and only 159 patients answered this question (59 patients were ≤40 y and 100 patients were >40 y). Twenty-nine women ≤40 y expressed a desire to have a child compared with ten women >40 y (49.15% vs. 10.0%, respectively) (*P *< 0.005) (Table 4).

**Table 4:** Patients' preferences for children post-treatment

Age at diagnosis	No. of patients	Desire for children	*P*-value
≤40 y	59	29	< 0.005
>40 y	100	10	

## Discussion

Adjuvant chemotherapy has improved the disease-free survival and overall survival of younger patients with breast cancer, however, the adverse effects induced by adjuvant chemotherapy have not been well studied. In this retrospective report, we specifically evaluate CIA following administration of adjuvant chemotherapy and endocrine therapy to patients diagnosed with breast cancer prior to the onset of menopause. When the patients were divided into two groups based on age to evaluate CIA, age was identified as an important predictor for CIA. This result is consistent with previous studies where CIA was observed to be more frequent in older women due to a reduced number of active ovarian follicles present [14]. In this study, rates of CIA were found to be higher in patients older than 40 y. It has been hypothesized that ovarian suppression may increase the efficacy of chemotherapy [15], but it is not clear whether CIA itself has an impact on prognosis or whether it is a marker of higher bio-availability causing both ovarian destruction and tumor-cell destruction [12].

Many clinical trials have found that the addition of taxane to anthracycline-based chemotherapy regimens prolongs the disease-free survival and overall survival of node-positive breast cancer patients. Early in 1994, navelbine was reported as a first-line therapy for the treatment of advanced breast cancer [16], and both docetaxel and navelbine have become widely used in the treatment of breast cancer. Previous studies showed that the incidence of CIA in taxane-based chemotherapy regimens was higher in anthracyclines-based chemotherapy regimens [17-19]. In a study by Martin et al. [18], the incidence of CIA associated with a TAC (docetaxel plus doxorubicin and cyclophosphamide) regimen was higher than that for a FAC (fluorouracil plus doxorubicin and cyclophosphamide) regimen (*P *= 0.007). Similarly, in a study by Han et al. [17], a higher incidence of CIA was associated with patients receiving taxane-containing chemotherapy (*P *= 0.002). In a study by Minisini et al. [20], patients receiving taxanes have an increase risk of CIA, but these patients recover menstrual bleeding more frequently than patients not treated with taxanes. Despite these results, it remained unclear whether the use of taxanes increased the rate of CIA compared to the use of anthracyclines alone [21]. In this study, the incidence of CIA for the FEC regimen was higher than that for the NE regimen (*P *< 0.05), while no significant differences were found between FEC and TE regimens. It has been hypothesized that chemotherapy regimens including cyclophosphamide aggravate the incidence of CIA, and early studies reported higher cyclophosphamide doses were associated with higher rates of CIA [22,23]. In the PACS01 trial, the incidence of CIA between 3FEC/3D and 6FEC regimens were not found to be statistically different, although the 3FEC/3D treatment was found to induce a greater number of reversible amenorrhea cases than 6FEC [24]. Similarly, in this study, more patients treated with TE regimen experienced resumption of menstruation after temporary amenorrhea (data not shown). An NE regimen including epirubicin at 50 mg/m^2 ^was previously included in the FASG trials [25], however, the incidence of CIA associated with this NE regimen alone was not reported. In this study, patients that received the NE regimen exhibited a lower incidence of CIA than the other two regimens. Our results further indicated that navelbine affected the incidence of CIA less than docetaxel, but no significant differences were found. It will be important for additional patient cases to be analyzed to confirm the results of this study.

Many studies have reported that administration of tamoxifen following chemotherapy increases CIA [26,27], however, the IBCSG trial, 13-93, found no statistically significant difference in the rate of CIA between patients that received tamoxifen or not [28]. In this study, tamoxifen use was found to be an important predictive factor for CIA, and the incidence of CIA was higher for patients treated with tamoxifen than for those who did not receive tamoxifen. These results are consistent with studies that demonstrated tamoxifen use contributed to CIA, especially when taken over a long period of time, and tamoxifen has been shown to delay the resumption of menses. Tamoxifen is additionally prescribed if patients are positive for ER and/or PR, and it results in ovaries stimulation [29]. So tamoxifen can cause increase in follicle stimulating hormone (FSH), but also an increase in estradiol. In fact, there was no difference in Anti Müllerian Hormone between users and no-users of tamoxifen suggesting that only growing follicles were influenced by tamoxifen [30]. These studies suggest that tamoxifen may be responsible for amenorrhea but not for ovarian failure.

The diagnosis and treatment of breast cancer often poses a threat to a patient's fertility, yet an increasing number of young women are concerned with maintaining their fertility in order to have children after a diagnosis of breast cancer. For patients treated for early-stage breast cancer, pregnancy following their treatment was not found to adversely affect their prognosis [10]. However, for the vast majority of women who remain amenorrheric after 1 y of treatment, they will not regain ovarian function, resulting in loss of child-bearing potential [31]. Therefore, options to preserve fertility should be considered as early as possible if patients convey their preference for becoming pregnant after breast cancer treatment. Unfortunately, less than half of oncologists discuss the possibility of treatment-related infertility with their patients [32]. Options that are available to preserve fertility include the use of GnRH agonists, cryopreservation of resected ovarian tissue, collection of fertilized or non-fertilized eggs after stimulation and puncture, or preservation of embryos that have undergone *in vitro *fertilization. However, it is important to note that the success of these options in maintaining women's fertility post-treatment is uncertain [33], and currently, there are no evidence-based recommendations for the preservation of fertility or ovarian function in breast cancer patients [34]. However, oncologists still have a responsibility to their patients to address concerns of fertility preservation when prescribing treatment regimens for younger breast cancer patients.

## Conclusions

In conclusion, an evaluation of clinical data from 170 premenopausal women diagnosed with breast cancer to evaluate rates of CIA was performed, and the type of chemotherapy regimen administered was found to affect the rate of CIA. Although docetaxel and navelbine were not associated with higher rates of CIA, cyclophosphamide appeared to contribute to much higher rates of CIA. Furthermore, patient age and tamoxifen were both found to significantly increase rates of CIA. We suggest that the results of this study and other clinical trials need to be considered when patients are concerned with preserving fertility following treatment for breast cancer.

## Abbreviations

CIA: chemotherapy-induced amenorrhea; FEC: fluorouracil plus epirubicin and cyclophosphamide; TE: docetaxel plus epirubicin; NE: navelbine plus epirubicin; LHRH: luteinizing hormone releasing hormone; NCCN: National Comprehensive Cancer Network; G-CSF: granulocyte colony stimulating factor; ER: estrogen receptor; PR: progesterone receptor; SD: standard deviation; IDC: infitrating ductal carcinoma; FSH: follicle stimulating hormone

## Competing interests

The authors declare that they have no competing interests.

## Authors' contributions

WBZ was responsible for analysis of data, as well as drafting the manuscript. HY was responsible for one center (**Nanjing Maternity and Child Health Care Hospital of Nanjing Medical University**). XAL, XMZ and Lin Chen were responsible for the other center (**The First Affiliated Hospital of Nanjing Medical University**). JCD performed the statistical analysis. ADT was responsible for the collection of most data. JJM was responsible for the collection of data of patients treated in her hospital. SW was responsible for the design of this study. Ling Chen and LJL were responsible for the collection of data and were involved in the analysis and interpretation. All authors read and approved the final manuscript.

## Pre-publication history

The pre-publication history for this paper can be accessed here:

http://www.biomedcentral.com/1471-2407/10/281/prepub
